# Two Uncommon Causes of Guillain-Barré Syndrome: Hepatitis E and Japanese Encephalitis

**DOI:** 10.1155/2015/759495

**Published:** 2015-12-22

**Authors:** Dhrubajyoti Bandyopadhyay, Vijayan Ganesan, Cankatika Choudhury, Suvrendu Sankar Kar, Parthasarathi Karmakar, Vivek Choudhary, Prasun Banerjee, Debarati Bhar, Adrija Hajra, Manas Layek, Sabyasachi Mukhopadhyay

**Affiliations:** ^1^Department of Accident and Emergency, Lady Hardinge Medical College, New Delhi, India; ^2^Department of Internal Medicine, R. G. Kar Medical College, Kolkata, India; ^3^Department of Internal Medicine, IPGMER, Kolkata, India; ^4^Lady Hardinge Medical College, New Delhi, India; ^5^R. G. Kar Medical College, Kolkata, India

## Abstract

We are presenting two cases of Guillain-Barré syndrome where it is preceded by hepatitis E virus (HEV) and Japanese encephalitis virus (JEV) infection, respectively. Our first case is a forty-three-year-old nondiabetic, nonhypertensive female who was initially diagnosed with acute HEV induced viral hepatitis and subsequently developed acute onset ascending quadriparesis with lower motor neuron type of bilateral facial nerve palsies and respiratory failure. Second patient was a 14-year-old young male who presented with meningoencephalitis with acute onset symmetric flaccid paraparesis. After thorough investigations it was revealed as a case of Japanese encephalitis. Our idea of reporting these two cases is to make ourselves aware about this potential complication of these two common infections.

## 1. Introduction

In about 70% of cases, Guillain-Barré syndrome (GBS) is preceded by an infection, most frequently by* Campylobacter jejuni (20 to 30%)*, but other pathogens, such as viruses from Herpesviridae family (*Cytomegalovirus*, varicella-zoster virus, and Epstein-Barr virus) or bacterial agents (*Haemophilus influenzae*,* Mycoplasma pneumoniae*), can be responsible in varying percentages [1]. Neurologic symptoms have been reported in up to 5% of patients with an HEV infection and in a study from Bangladesh, HEV IgM was positive in 11% of GBS patients. Most of the patients were immunosuppressed [2]. These data indicate the neurotropism potential of HEV. Japanese encephalitis (JE) is the most important cause of acute and epidemic viral encephalitis. JE is mainly prevalent in children and adolescents below 20 years of age. JEV is also known to cause varieties of neurological complications. GBS induced by hepatitis E virus and JEV are extremely rare.

## 2. Case Presentation

### 2.1. Case Number 1

A 43-year-old female presented with history of fever for three days which was soon followed by yellowish discoloration of urine, loss of appetite, and nausea. Two weeks after the onset of fever she noticed ascending type of weakness involving lower limbs followed by upper limbs and then developed difficulty in swallowing both solid and liquid diets. She also noticed gradually progressive hoarseness of voice, respiratory difficulty, and inability to close her eyes.

Examination revealed presence of icterus and showed progressive decline of single breath count over days. Neurological examination showed lower motor neuron type of bilateral seventh, ninth, and tenth cranial nerves palsies with quadriparesis. All the four limbs were flaccid, areflexic with motor power being grade 4/5 and grade 2/5 in upper and lower limbs, respectively. Superficial reflexes including plantar were absent bilaterally. The rest of the central nervous system examinations were within normal limit. On abdominal examination there was soft tender hepatomegaly.

Laboratory investigations revealed normal hemogram with liver function test showing total bilirubin 5 mg/dL (normal 0.3–1.3 mg/dL), conjugated bilirubin 3 mg/dL (normal 0.1–0.4 mg/dL), AST 1080 U/L (normal range 12–38 U/L), ALT 1950 U/L (normal range 7–41 U/L), ALP 350 U/L (normal range 50–160 U/L), total protein 7 gm/dL (normal 6.7–8.6 g/dL), and albumin 4 gm/dL (normal 3.5–5.5 g/dL) typically suggestive of acute viral hepatitis. In etiological search of viral hepatitis we found HEV IgM was highly positive (EIAgen HEV IgM, Adaltis, Spain, with 98 percent sensitivity and specificity) with negative anti-HEV IgG and RT-PCR (performed by RT-PCR kit, Qiagen GmbH, Hilden, Germany) for HEV was positive in the serum sample taken on the day of admission. On nested broad-spectrum reverse transcription PCR with amplification within the open reading frame (ORF) 1 region of HEV genome revealed HEV genotype 3. HBsAg, anti-HCV antibody, IgM anti-HAV antibody, and anti-HIV antibody were negative. Both IgG and IgM were negative for Epstein-Barré virus (EBV), herpesvirus, and adenovirus. Serology for* Campylobacter*, varicella-zoster virus, and* Cytomegalovirus* (CMV) was negative. Prothrombin time and activated partial thrombin time were normal.

Study of cerebrospinal fluid showed albuminocytological dissociation with albumin being 350 mg/dL (protein 450 mg/dL) (normal range of protein 15–45 mg/dL) and total cell count 12/cmm (all lymphocytes) (normal value 0–5/cmm), glucose 65 mg/dL (normal > 60 mg/dL), and ADA 1.2 U/L. CSF was positive for anti-HEV IgM but negative for anti-HEV IgG. CSF was also negative for HEV RNA, CMV DNA, EBV DNA, and varicella-zoster virus (VZV) DNA.

Nerve conduction velocity (NCV) study suggested demyelination and axonal neuropathy. Meanwhile anti-ganglioside antibody (GM1) came out to be positive.

Temporal profile of clinical condition and relevant investigations of this case led us to clinch the diagnosis as Guillain-Barré syndrome with respiratory failure due to preceding HEV hepatitis.

Three days after admission patient developed autonomic instability in the form of fluctuation of blood pressure to a great extent. Patient developed progressive respiratory difficulty and single breath count is 12 per breathing. Then the patient was transferred to the ICU and was put on invasive ventilator. Once investigations were suggestive of GBS, intravenous immunoglobulin (IVIG) was administered at a rate of 0.4 gm/kg/day for five days. With supportive therapy patient showed signs of gradual improvement and after seven days ventilator could be weaned successfully. Rehabilitation programme initiated side by side. At the time of submission of this paper, patient could manage to stand independently and walk a few steps with support.

### 2.2. Case Number 2

A 14-year-old young male presented with low grade fever, headache, vomiting, and myalgia. Three days later he developed sudden onset symmetric quadriparesis with difficulty in rolling over the bed. The patient had no history of difficulty in urination, defecation, or band-like sensation in the abdomen.

Examination revealed hypotonia and absent deep tendon reflexes with power 2/5 in all the four limbs. The plantar response is bilaterally nonresponsive. Signs of meningeal irritation like neck rigidity and Kernig's sign were present. Higher functions, cranial nerve examinations, sensory examination, autonomic functions, and cerebellar functions were within normal limits.

**Figure 1:**
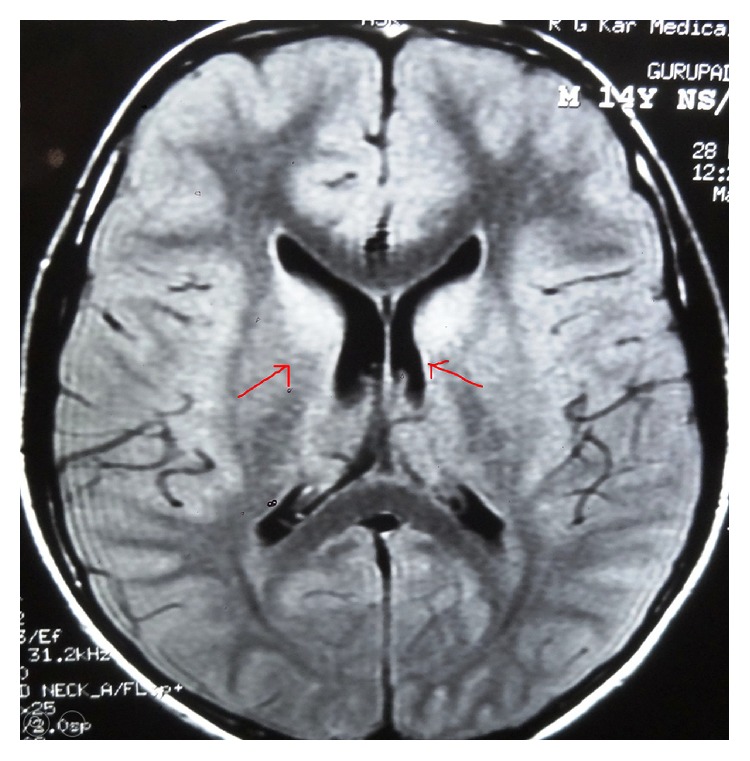
MRI (T2 FLAIR) showing hyperintensities of both caudate nuclei.

Laboratory investigations showed normal hemoglobin%, total count 10800/cmm (N_85_L_10_M_3_E_2_), and normal renal and liver function tests. Blood reports for dengue, malaria, and anti-HIV antibody were negative. CSF study revealed cells count 330/cmm with lymphocytes 88% and polymorphs 10%, sugar 67 mg%, and protein 147 mg%. MRI brain (T2 FLAIR) showed hyperintensities in both the caudate nuclei and thalami (Figure 1). Since the MRI findings were suggestive of Japanese encephalitis, CSF and serum were tested for IgM anti-JEV using IgM antibody capture (MAC) ELISA kit supplied by the National Institute of Virology, Pune. Both came out positive for Japanese encephalitis virus (IgM antibody by ELISA method) infection. Polymerase chain reaction (PCR) for* Mycobacterium*, herpesvirus, varicella-zoster virus,* Enterovirus*, and rabies virus was negative. Serology for herpesvirus, HAV, HEV, CMV, EBV, dengue virus, HIV,* Leptospira* species, and* Rickettsia* species was negative. Blood and CSF cryptococcal antigen and malaria blood smear were also negative. NCV study of all limbs showed demyelinating neuropathy.

The patient was on intravenous acyclovir for 3 days before being referred to our Institute. After thorough investigation it appeared to be a case of possible Japanese encephalitis. Acyclovir was stopped and IV immunoglobulin was initiated at a rate of 0.4 gm/kg/day for five days. Next day he developed respiratory distress with respiratory rate of 34/min and single breath count is 12 per breathing. The patient was shifted to the ICU and was put on invasive ventilator. The patient developed two episodes of GTCS in the ICU and was managed with midazolam and thiopental sodium infusions. The patient improved gradually and was weaned from ventilator on 7th day. Physiotherapy was advised during discharge for his residual weakness of the limbs. At the time of discharge patient can walk with support.

## 3. Discussion 

Hepatitis E virus is one of the most common causes of acute hepatitis worldwide, with the majority of cases occurring in Asia. In recent years, however, an increasing number of hepatitis E virus infections have been reported in industrialized countries which are being considered as autochthonous (indigenous) infection. The importance of this infection resides in the associated morbidity and mortality which may be related to extrahepatic complications including nervous system [3]. Between 2004 and 2009, up to 5.5% of neurologic complications were reported in a series of 126 patients of HEV hepatitis in two hospitals in the United Kingdom and France; only one case was consistent with the diagnosis of GBS. Data about neurologic sequelae of HEV infection are scarce and come mainly from the Indian subcontinent with HEV genotype 1. Recently an increased number of autochthonous HEV infection (mainly genotype 3) with GBS have been reported from developed countries [4]. An increased ratio of anti-HEV immunoglobulin (Ig) M antibodies was found in 10 patients with GBS (5.0%) compared with 1 healthy control (0.5%, odds ratio 10.5, 95% confidence interval 1.3–82.6, *p* = 0.026) in a study in Netherlands. HEV RNA was detected in blood from 3 of these patients [5]. The neurological complications associated with HEV reported so far were inflammatory polyradiculopathy, bilateral brachial neuritis, encephalitis, Guillain-Barré syndrome, and ataxia/proximal myopathy [6]. GBS is a rare neurological complication in relation to HEV hepatitis. Bilateral facial palsy which is seen in our patient is very uncommon.

The GBS is considered to be an autoimmune disease triggered by a preceding bacterial or viral infection. A molecular mimicry mechanism is supposed to be involved in the pathogenesis. The nature of the epitope, although still uncertain, is likely to be a glycolipid. The most attractive targets are gangliosides, which are present in nodal and internodal membranes of nerve fibres. Ganglioside antibodies may perturb nerve conduction and, in a complement-dependent fashion, disrupt the molecular topography of nodal and paranodal proteins and induce motor axonal degeneration. It is postulated that infected cells can produce ganglioside-like epitopes that trigger the immune response [7]. The most commonly identified triggering agents are* Campylobacter jejuni*, followed by* Cytomegalovirus*, Epstein-Barr virus, and* Mycoplasma pneumoniae*. HIV,* Shigella*,* Clostridium*,* Haemophilus influenzae*, and hepatotropic virus like hepatitis A, hepatitis B, and hepatitis C were also identified as triggering agents [8]. But worldwide most commonly occurring HEV hepatitis is yet to be well documented initiating viral infection in GBS. There was also a study performed in Bangladesh, which confirmed the relation between GBS and HEV [9]. In our case, the temporal association between acute hepatitis E and GBS strongly suggested a relation between both disorders.

Japanese encephalitis (JE) is the most important cause of viral encephalitis in Asia. JE is caused by Japanese encephalitis virus (JEV), a mosquito-borne virus, belonging to the genus* Flavivirus* (family Flaviviridae). WHO estimated that approximately 67 900 JE cases typically occur annually in the 24 JE-endemic countries, for an incidence of 1.8 per 100 000 overall [10]. Pathological changes observed in humans and animals with JE include (1) neuronal and glial damage directly caused by viral replication; (2) inflammation, including perivascular infiltration of small lymphocytes, plasma cells, and macrophages; (3) cellular nodule formation composed of activated microglia and mononuclear cells; and (4) cerebral interstitial edema [11]. The important presenting features of JE in a previous large case series were altered sensorium, convulsions, headache, hyperkinetic movement disorders, features of brain stem involvement as opsoclonus, gaze palsies and pupillary changes, dystonia, decerebrate rigidity, paralysis, and seizures [12]. Unusual features of JE described in the literature include respiratory paralysis, oromandibular dystonia, hemiplegia with dysarthria, and acute flaccid paralysis [13].

## 4. Conclusion

The actual incidence of GBS associated with HEV and JEV infection is unknown because most of the hepatitis E and JEV are still underdiagnosed. This is in part due to the fact that frequently these infections are subclinical. Hence, HEV and JEV infection should be considered in GBS patients associated with abnormal levels of liver enzymes and higher neurological function alteration, respectively.
